# It is time to celebrate the importance of evaluation in medical education

**DOI:** 10.5116/ijme.5aed.6f12

**Published:** 2018-06-04

**Authors:** John Sandars

**Affiliations:** 1 Postgraduate Medical Institute (PGMI), Faculty of Health & Social Care, Edge Hill University, UK

**Keywords:** Evaluation, medical education, UK

## Introduction

An ever-increasing volume of research studies are being submitted by medical educators for publication and also as presentations at seminars, symposia, and conferences. An important concern is the extent to which the findings from these research studies are being used by medical educators to inform their decision-making about future policy and practice. The simple answer appears to be that there is limited use of many medical education research studies to inform decision-making. For example, a recent study on the translation of research findings by medical educators has highlighted the frequent lack of useful knowledge from research to support their decision –making, with medical educators requesting more information about the process of an intervention (how did it work?) in addition to the outcome (did it work?).^1^

In this editorial, I will challenge the importance that is currently attached to research in medical education and propose a greater emphasis on high quality evaluation to provide the essential useful knowledge for decision-making. This change in emphasis has major implications for the future of medical education scholarship, with its current focus on the ‘research scientist’ role in the demonstration of scholarship.

### Importance of ‘useful knowledge’

A wide variety of stakeholders, from strategic policymakers to grass-roots teachers, have to make important decisions about how medical education is delivered and supported, especially whether to implement a particular intervention or not. This decision-making process requires ‘useful knowledge’.^2^ The concept of useful knowledge was first used to describe the observation that many of the findings from organisational research were not being used to inform policy or practice. It was noted that decision-makers required greater understanding of the factors that influenced the variation of outcomes across different contexts and that the research did not provide this essential useful knowledge. This observation has major implications for all researchers in the way that they conduct research, including medical education. As an example of the lack of useful knowledge, few research studies on the use of technology in medical education appear to report the essential usability aspects (with a focus on the ease of use). However, this information about usability is essential if the intervention is to be used more widely in other contexts.^3^

### Limitations of research for useful knowledge

Medical education research studies can be classified into description studies (with a focus on what was done?), justification studies (with a focus on did it work?), and clarification studies (with a focus on how did it work?).^4^   The interest in evidence–based medical education, with its concern about the outcomes of ‘what works and does not work’, appears to have led to increasing numbers of justification research studies being performed in medical education and presented for publication and conferences.However, there has been a relative lack of clarification studies that provide the essential useful knowledge for decision-making. This situation is highlighted in a recent study of 1398 conference abstracts presented at four major international simulation based medical education conferences, in which 36.3% were justification studies but only 9.3% were clarification studies.^5^

Medical educators do require useful information on outcomes, but concerns have been raised about the quality of many justification research studies. There are several major difficulties in performing ‘gold standard’ experimental studies in medical education,^6^ and there are also methodological limitations in the commonly used pre-post intervention studies, whether limited to the same cohort or with a comparison between cohorts.^7^ In the wider educational research literature, concerns have also been raised about the different outcomes noted in replication studies and the publication bias towards not publishing this type of study, especially if the intervention is noted to be less effective in another context.^8^

### Research or evaluation in medical education

The emphasis on performing research by medical educators appears to be related to the increasing calls to have a ‘research scientist’ role in the demonstration of their medical education scholarship.^9^ However, the wider literature on educational scholarship calls for a ‘systematic inquiry’ into practice, with a focus on the explicit and rigorous use of methods (including data collection and analysis) to understand practice.^10^^,^^11^ This  approach to educational scholarship appears to be more concerned with the quality of  how the study has been performed and less concerned with conceptual differences between research and evaluation.  In medical education, research has been described as having a focus on producing generalisable results that can be published in peer reviewed literature and requires ethical approval.^12^ This is in contrast to evaluation in medical education, which has a focus on quality-improvement and carried out for local use.^12^  This conceptual difference,  and the implied relative merit of research compared with evaluation,  appears to be important in medical education, probably since it aligns with the definitions used for clinical and health services research and evaluation studies.

### Celebrating evaluation in medical education

My own personal experience is that many medical educators are mostly engaged with evaluation and not research, so why not celebrate evaluation? My proposal is that medical educators should consider evaluation as an essential aspect of their scholarship and that a future direction for medical education scholarship is to engage in high-quality evaluation by developing an increased understanding of the science of evaluation.^13^   An evaluation has an intention to make a judgment about the value or worth of an intervention.^13^ This definition highlights the importance of evaluations in providing useful knowledge for decision-making, with information about both outcome (did it work?) and process (how did it work?).The information that is provided also has to be credible for it to be useful knowledge and it is essential to ensure that a rigorous systematic approach is applied to evaluation and that peer-review opportunities are provided though wider dissemination by submission for publication and presentations at conferences.

Performing high-quality evaluation in medical education should be the vision for all medical educators. Two approaches can achieve this vision. First, it is essential to have detailed and explicit reporting of findings that contain the essential details of both the outcome and the process. Currently, an adaptation of the SQUIRE (Standards for Quality Improvement Reporting Excellence) guideline with a specific focus on medical education is being developed, but the existing guideline covers the main aspects and can provide a template for presenting the findings of an evaluation.^14^ Second, it is essential that data collection and analysis is rigorous.^15^ This requires clear adherence to the principles of ‘best practice’ for quantitative and qualitative research methods. A major criticism of evaluation is that the findings are related to a specific context, and that generalisation or transferability to other contexts is low.^15^ However, there is a similar argument for a single site or single cohort research studies. A strength of any high-quality evaluation is that it is a case study, providing a rich and detailed description of a case, which includes context, processes, and outcomes.^16^ However, the findings of a single case study can have wider application if there is a synthesis of several case studies from different contexts or a single case study becomes ‘instrumental’, linking the findings to the broader literature or to an underlying theory.^17^

## Conclusions

There is a need for justification research studies to inform both policy and practice decision-making, but high-quality justification research requires methodological expertise to develop, perform, analyse and present findings that have the necessary rigour. Similar to clinical and health services research, multi-disciplinary research groups with specific expertise are likely to be required to produce medical education research that can provide the high-quality justification studies demanded by adopting an evidence-based perspective to decision-making. However, most medical educators will not have the opportunity to join these research groups.

My plea for celebrating evaluation may be radical but most medical educators appear to currently focus on evaluation instead of research. I believe that if evaluation can be high quality then it is worthy of being published in major medical education journals and presented at national and international conferences. This dissemination of findings is essential if useful knowledge is to be made available to all stakeholders and is also an essential aspect of medical education scholarship.

### Conflicts of Interest

The author declares that they have no conflict of interest.
